# Clinical Applications of Three-Dimensional Printing in Upper Extremity Surgery: A Systematic Review

**DOI:** 10.3390/jpm13020294

**Published:** 2023-02-06

**Authors:** Andrzej Hecker, Lukas Tax, Barbara Giese, Marlies Schellnegger, Anna-Lisa Pignet, Patrick Reinbacher, Nikolaus Watzinger, Lars-Peter Kamolz, David Benjamin Lumenta

**Affiliations:** 1 Research Unit for Digital Surgery, Division of Plastic, Aesthetic and Reconstructive Surgery, Department of Surgery, Medical University of Graz, 8036 Graz, Austria; 2 Division of Plastic, Aesthetic and Reconstructive Surgery, Department of Surgery, Medical University of Graz, 8036 Graz, Austria; 3 COREMED-Centre for Regenerative Medicine and Precision Medicine, Joanneum Research Forschungsgesellschaft mbH, 8010 Graz, Austria; 4 Medical University of Graz, 8036 Graz, Austria; 5 Department of Orthopaedics & Traumatology, Medical University of Graz, 8036 Graz, Austria

**Keywords:** 3D printing, upper extremity, rapid prototyping, patient-specific

## Abstract

Three-dimensional printing for medical applications in surgery of the upper extremity has gained in popularity as reflected by the increasing number of publications. This systematic review aims to provide an overview of the clinical use of 3D printing in upper extremity surgery. Methods: We searched the databases PubMed and Web of Science for clinical studies that described clinical application of 3D printing for upper extremity surgery including trauma and malformations. We evaluated study characteristics, clinical entity, type of clinical application, concerned anatomical structures, reported outcomes, and evidence level. Results: We finally included 51 publications with a total of 355 patients, of which 12 were clinical studies (evidence level II/III) and 39 case series (evidence level IV/V). The types of clinical applications were for intraoperative templates (33% of a total of 51 studies), body implants (29%), preoperative planning (27%), prostheses (15%), and orthoses (1%). Over two third of studies were linked to trauma-related injuries (67%). Conclusion: The clinical application of 3D printing in upper extremity surgery offers great potential for personalized approaches to aid in individualized perioperative management, improvement of function, and ultimately help to benefit certain aspects in the quality of life.

## 1. Introduction

**Figure 1:**
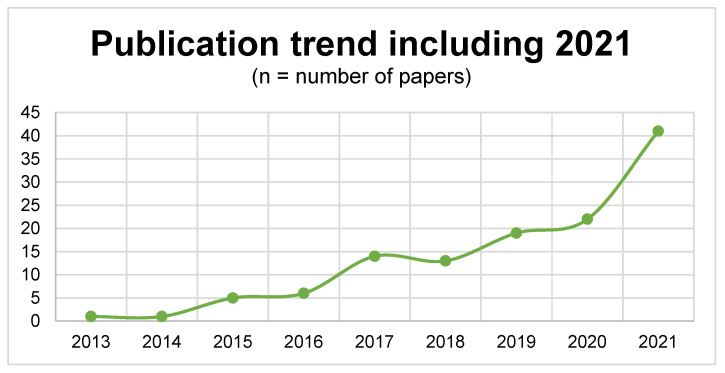
Number of publications relating to 3D printing in upper extremity surgery by year. In *PubMed*, the following search query was used: (“3D” OR “3-dimensional”) AND “print*” AND (“upper extremity” OR “upper limb”).

In upper extremity surgery, numerous types of implants are used for bone fixation or arthroplasty, and may ideally fit the specific region [1]. The high cost of functional prostheses, the duration of production, as well as the highly complex manufacturing process not only limit its accessibility but also affect medical research and development [2]. Three-dimensional (3D) technology was first introduced by Chuck Hill as stereolithography in 1986. The majority of medical applications, requiring a 3D image (computed tomography scan or magnetic resonance imaging scan) in a digital imaging and communications in medicine (DICOM) format, later converted to an STL format, were used for stereolithography computer-aided manufacturing [1,3]. To reduce costs and complexity, new technologies and formats were introduced, notably during the commercialization of the 3D-printing technology [2]. The technological progress in 3D printing did of course not spare the clinical sector [4], where the technology has been used for preoperative planning, production of orthoses and prostheses, intraoperative modeling of autologous bone grafts, and for training purposes [5]. Especially in the past five years, the use of 3D printing of upper extremity prostheses has developed tremendously. Despite its increasing use in medicine, no specific design guidelines exist, and an overview of clinically available 3D-printed tools is missing. The discussion of the benefit of 3D printing for clinical application is ongoing [6]. While some studies highlight advantages in patient-specific fit accompanied by lower manufacturing costs, no assembly and material waste, and reduced operating time [1], other studies did not report any significant advantages using 3D printing compared to conventional approaches [7,8]. During the past decade, the medical application of 3D-printing technology, especially for upper extremity surgery, has gained traction as reflected in the increasing publication trend of recent years (Figure 1). The aim of this systematic review was to identify clinical studies that demonstrate a direct clinical use of 3D printing in patients with upper extremity injuries or deformities and provide an overview of its applications.

## 2. Materials and Methods

### 2.1. Study Protocol

The systematic review and its methods were approved and registered on the International Prospective Register of Systematic Reviews (PROSPERO) under the protocol number CRD42021247588. This study was conducted and reported in accordance with the Preferred Reporting Items for Systematic Reviews and Meta-Analyses statement [9].

### 2.2. Search Strategy

A literature search was performed in the online databases *PubMed* and *Web of Science* using the following search term strategy:

(“3D-print*” OR “3-dimensional print*” OR “three-dimensional print*” OR “rapid prototyping” OR “additive manufacturing” OR “computer-aided design” OR “bioprinting” OR “biofabrication”) AND (“upper limb” OR “upper-limb” OR “upper-extremity” OR “upper extremity” OR “hand” OR “hands” OR “wrist” OR “finger” OR “fingers” OR “phalange” OR “phalanges” OR “digit” OR “digits”).

To minimize the risk of missing relevant data, additionally, the following MeSH term has been used in the *PubMed* database:

(“Printing, Three-Dimensional” [Mesh]) AND (“Upper Extremity” [Mesh] OR “Upper Extremity Deformities, Congenital” [Mesh] OR “Hand Injuries” [Mesh] OR “Hand” [Mesh] OR “Hand bones” [Mesh] OR “Fingers” [Mesh] OR “Finger Phalanges” [Mesh] OR “Bones of Upper Extremity” [Mesh] OR “Arm Bones” [Mesh] OR “Arm” [Mesh] OR “Artificial Limbs” [Mesh] OR “Forearm” [Mesh] OR “Wrist” [Mesh] OR “Wrist Joint” [Mesh] OR “Forearm” [Mesh] OR “Wrist” [Mesh] OR “Wrist Joint” [Mesh] OR “Shoulder” [Mesh] OR “Elbow Joint” [Mesh] OR “Elbow” [Mesh] OR “Elbow Prosthesis” [Mesh] OR “Hand Joints” [Mesh] OR “Finger Joint” [Mesh]).

### 2.3. Article Selection

The search was conducted in August 2021 for studies written in the English or German language with no restrictions with regard to publication year. Two authors (L.T. and B.G.) independently screened titles, abstracts, and available full articles identified in the online databases *PubMed* and *Web of Science*. Studies considered for inclusion were clinical studies that described direct clinical applications of 3D printing. Studies that did not investigate direct clinical applications, such as simulation studies and technique development, were excluded. Articles including experimental studies involving laboratory studies, cadaver studies, and examinations on animals, reviews, commentaries, or letters were also excluded.

The reviewers (L.T. and B.G.) recorded all search results with regard to study characteristics, clinical entity, type of clinical application, concerned anatomical structures, reported outcomes, and level of evidence (American Society of Plastic Surgeons (ASPS) Evidence Rating Scale for Therapeutic Studies [10]) in an Excel sheet (Microsoft Excel 2016 Microsoft Office (16.44) 32-bit). Results were independently compared and reconciled. If a discrepancy occurred between the reviewers, the articles were evaluated by a third reviewer (A.H.).

## 3. Results

### 3.1. Literature Search

**Figure 2:**
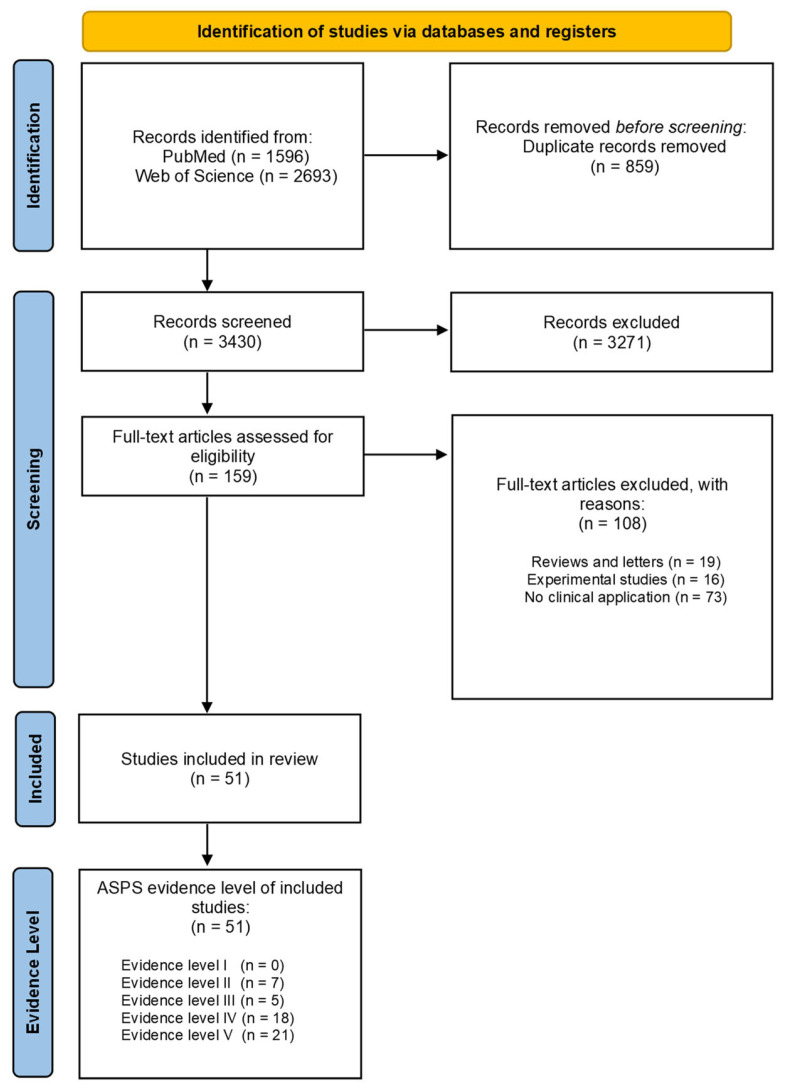
Modified PRISMA Flow Diagram. PRISMA: Preferred Reporting Items for Systematic Reviews and Meta-Analyses. ASPS: Evidence Rating Scales of the American Society of Plastic Surgeons.

The initial literature search yielded in total 4289 studies, 1596 studies in *PubMed* (thereof 171 studies from MeSH terms) and 2693 studies in *Web of Science.* After duplicate exclusion (859 duplicates), the remaining 3271 studies of the 3430 studies in total were excluded due to nonapplicability of the exact study purpose. Afterwards, titles, abstracts, and, if not unambiguous, full-text articles of the remaining 159 studies were analyzed concerning the inclusion and exclusion criteria. Articles were excluded according to the previously mentioned exclusion criteria including reviews or letters/commentaries (n = 19), experimental studies (n = 16), and no clinical application (n = 73). Based on our inclusion and exclusion criteria, 51 clinical studies have been found that demonstrate a clinical application of 3D printing in patients with upper extremity injuries or deformities (Figure 2).

In the following section, results of study characteristics, entities and application for which 3D printing was utilized, involved anatomical upper extremity structures, reported outcomes, outcome results of comparative studies, and evidence level of all 51 included studies are summarized descriptively.

### 3.2. Study Characteristics

**Figure 3:**
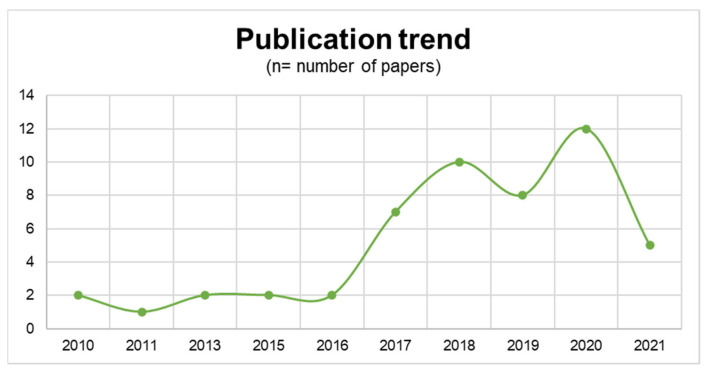
Number of included studies by year. Data from 2021 do not include the full year as the search was performed in August 2021.

The 51 included studies were published between 2010 and 2021. There was a trend towards an increasing number of publications in more recent years, with 9 studies published from 2010–2016, and 42 studies published from 2017–2021 (Figure 3). There were a total of 355 patients included in this study. For the 293 patients for which gender was specified, 115 were females and 178 were males. The mean age of included patients was 36.6 years (range, 3–79 years). The majority of included studies were case reports (n = 23) followed by case series (n = 16), randomized controlled trials (RCTs) (n = 7), retrospective cohort studies (n = 3), and prospective cohorts (n = 2).

### 3.3. Entities

The entities (n = number of studies, N = number of patients) in which 3D printing was utilized were trauma-related injuries (n = 34, N = 248), carcinoma (n = 7, N = 33), followed by bone deformities (n = 6, N = 33), congenital anomalies (n = 3, N = 23), and osteonecrosis (n = 2, N = 6), overuse syndrome (n = 1, N = 11), infection (n = 1, N = 1).

### 3.4. Applications

The most common procedures in which 3D printing was utilized were patient-specific support for intraoperative use (n = 17), body implants (n = 15), followed by preoperative planning (n = 14), prostheses (n = 8), and orthoses (n = 4).

### 3.5. Involved Anatomical Upper Extremity Structures

The most common anatomical upper extremity structure for which 3D printing was utilized was the radius (n = 19, N = 180), followed by humerus (n = 11, N = 81), ulna (n = 11, N = 25), carpal bones (n = 8, N = 42), scapula (n = 5, N = 10), phalanges including thumb (n = 7, N = 11), and clavicle (n = 3, N = 3).

### 3.6. Reported Outcomes

The reported outcomes included function (n = 42), pain (n = 26), strength (n = 17), satisfaction (n = 13), operation duration (n = 10), intraoperative blood loss (n = 5), and communication between patient and surgeon (n = 1).

### 3.7. Comparative Outcome Results (3D-Printed Group vs. Conventional Group)

A total of 12 comparative studies were included. Out of these, eight investigated operation time, five intraoperative blood loss, three pain relief, and seven investigated functional outcomes. Table 1 and Table 2 illustrate an overview of the comparative results between the 3D-printed group and the conventional group. In all eight studies that investigated operating time, a significant reduction was shown in the 3D-printed group. A significant reduction of intraoperative blood loss in the 3D-printed group was observed in all five studies. None of the studies reported a significant difference in pain relief between conventional and 3D-printed groups. Out of seven, three studies on functional improvement reported a beneficial outcome in the 3D-printed group, one reported a significant improvement in the conventional group, while the other studies found no significant difference.

### 3.8. Evidence Level

The 51 articles meeting the inclusion criteria were ranked according to their level of evidence: 21, 18, 5, 7, 0 studies for level of evidence V, IV, III, II, I, respectively [10].

### 3.9. Language/Nation of Affiliated Institution

One study was in German, the remaining 50 included studies in English. The majority of the included studies came from China (n = 17), followed by the USA (n = 7), Switzerland (n = 5), Japan (n = 3), Germany (n = 3), and Belgium (n = 3). Two studies came from Korea, Italy, Chile, respectively. One study came from France, Canada, Thailand, Norway, Spain, United Kingdom, Netherlands, respectively.

## 4. Discussion

### 4.1. Applications

Included publications of this review have described 3D printing in direct clinical applications of upper extremity surgery in the following areas: preoperative planning of models, aid for intraoperative use, and the production of body implants, orthoses, and prostheses for specific patient requirements. Clinically relevant results of comparative studies (without case reports/series) are summarized in Table 1 and Table 2. The following section describes the results of the 51 included publications according to the corresponding applications.

### 4.2. Preoperative Planning

A 3D-printing application in upper extremity surgery allows clinicians to manufacture 3D-printed anatomical models for preoperative planning; 27% (14 out of 51 studies) of all included studies utilized 3D printing for preoperative planning, whereby the majority were case reports or series (9 out of 14 studies). Four from a total of seven included RCTs addressing this application [11,12,13,14]. Their results suggest that by using 3D-printed anatomical templates of the respective affected bone of the upper extremity, operation time, blood loss, and application of intraoperative fluoroscopy can be reduced [11,12,13,14]. This was observed not only in fractures of the distal radius [12], radial head [11], coronoid process of the ulna [11], humeral condyle [11], and intercondylar fractures of the humerus [14], but also in complex intraarticular fractures [13]. These 3D-printed models were also used for preventing plate fixation in corrective osteotomy for a malunited upper extremity [22,23] or to form a cement spacer for endoprosthetic radius reconstruction [24]. However, none of these studies could show a difference between the 3D-printing group and conventional therapy group in regards to long-term functional outcomes and pain (follow-up range 6 to 16 months) [11,12,13,14]. Nevertheless, patients of the 3D-printing groups presented with good postoperative function and without increased postoperative complication rates. In syndactyly surgery, a custom-made 3D-printed silicon syndactyly model in combination with an incision pattern template was successfully used to plan desyndactylization procedures [25]. Although operation duration was not investigated in this study, the authors believe that preoperative 3D-print-assisted incision pattern planning can reduce operation duration [25]. Even in challenging and complicated thumb reconstructions with second toe transplant, using 3D-printed bone and joint models revealed shorter operation duration and satisfying functional outcomes [26]. None of these studies reported 3D-printed model-related adverse events. On the one hand, this technique seems to be safe and accurate in upper extremity surgery, while on the other hand, 3D printing seems to have no advantage in preventing postoperative complications compared to conventional therapy. Overlapping fracture patches and angles of fracture lines are common uncertain factors for the surgeon during operation. These factors can lead to longer operation duration, more blood loss, and uneven joint surface, leading to higher postoperative complication rates [27]. By using a 3D-printed fracture model, a multi-angle and all-round observation in 360° is possible. Thus, fracture lines, broken bone fragments, and their spatial position in relation to each other can be investigated.

Hence, accurate description of fracture characteristics is feasible, supporting surgeons to make an individual and reasonable plan for patients. In addition to preoperative planning, 3D-printed models allow simulation and training of complex personalized surgical procedures. This surgery-related simulation and training may account for the reduced operation duration, which may subsequently lead to reduced intraoperative bleeding [11,12,13,14]. Likewise, the 3D-printing approach decreased the frequency of intraoperative fluoroscopy [12,13,14], which ultimately leads to a reduced exposure to radiation both for surgeons and patients. Moreover, using 3D-printed fracture models, for example, in preoperation discussion, provides more effective communication between surgeons and patients. Patients in the 3D-printing group more likely understood their medical condition and the surgical procedure compared to the conventional treatment group [11,12,14]. Although 3D printing has several advantages in the application of preoperative planning, there are also limitations. Due to the fact that the 3D-printing technology is based on bone computer tomography (CT) images, there is a lack of information about the surrounding soft tissue and vasculature. Especially in severely comminuted fractures, 3D printing has its limits, as small fracture fragments below a size of 0.8 mm remain undifferentiated [14]. In addition, using 3D-printed models is not possible for emergency cases, and about 4 to 6 h from the CT scan to the 3D-printed anatomical model are required [11,14].

### 4.3. Intraoperative Support

Thirty-three percent of all included studies used 3D printing for intraoperative aids, whereby the majority were again case reports or series (twelve out of seventeen studies). Two prospective [17,28], two retrospective studies [15,18], and one RCT [16] addressed this application. The study by Yin et al. [16] conducted an RCT of 16 patients with scaphoid nonunion without displacement. In eight patients, arthroscopy-assisted nonvascularised bone graft and fixation were performed with a conventional freehand technique using intraoperative fluoroscopy to confirm the position of bone-stabilizing Kirschner wires (K-wires). For eight patients, a 3D-printed patient-specific guide plate system was additionally employed. The 3D-printed guide was used for predrilling K-wire holes to obtain an anatomically accurate and correct direction. The authors reported a significant difference in operation time, which was 94.1 min in the conventional group and 69.4 min in the 3D-printing-assisted group. While conventional treatment of a scaphoid nonunion with nonvascularised bone grafts can achieve union rates of 72.5% [29], all patients treated with a combination of carpal arthroscopy presented scaphoid union within 6 months. However, the use of 3D-printed guide plate systems showed more accurate results [16]. Here, it is worth mentioning that through the minimally invasive arthroscopic technique, the scaphoid blood supply might be better protected, which may result in a higher reunion rate [16,29]. Nevertheless, there was no difference between the conventional group and the 3D-printing group regarding the postoperative functional outcomes and pain 6 months postoperation [16]. A prospective study of 22 patients with displaced scaphoid fractures and nonunions showed a significant difference in average residual fracture displacement, which was 22° in the conventional group and 7° in the 3D-printed drill guide group. Additionally, 8 out of 9 scaphoids in the 3D-printed group showed sufficient healing after 2 to 6 months, while healing in 11 out of 13 scaphoids of the conventional group was reported to occur between 2 and 34 months [17]. These results indicate that the use of 3D-printed guides for scaphoid reconstructions leads to a better anatomical fit due to a higher accuracy compared to the conventional freehand technique. A retrospective study of 56 patients with posttraumatic diaphyseal forearm deformities demonstrated that the operating time was significantly reduced from an average of 140 min in the conventional group to 108 min in the 3D-printed group [18]. Zhang et al. [15] showed similar results in adolescent cubitus varus deformities. Moreover, the intraoperative blood loss was reduced in the 3D-printed group. In both studies, a patient-specific guide for predrilling screw holes and a cutting guide to guide the saw blade for the osteotomy were used. Twelve weeks after surgery, Bauer et al. reported a significantly better outcome in the 3D-printed group compared with the conventional group [18]. Of note, there was a smaller number of patients who required an open wedge osteotomy in the 3D-printed group (13 vs. 23). However, after 24, 36, and 52 weeks there was no significant difference concerning consolidation of the osteotomy [18]. Again, the 3D-printed group showed a comparable functional outcome [15,18]. Nevertheless, the average follow-up time was significantly longer in the conventional group (25.4 months) compared with the 3D-printed group (13.6 months), therefore long-term results should be interpreted with caution [18]. Nevertheless, the preoperative 3D-assisted planning is more time-consuming and costly. The authors estimated that planning 3D-assisted corrective osteotomy takes about 2–4 h per patient, including CT scans from the contralateral side, and an additional cost of USD 2.415 for planning and producing the patient-specific guide [18]. A prospective study of corrective osteotomy in 16 patients with heterogenic posttraumatic malunited fractures (distal radius, distal humerus, diaphyseal forearm) showed highly satisfying functional outcomes [28]. Of note, a control group was missing, thus the authors assumed a residual deformity within 10°, which is an acceptable range in clinical practice. Fifty-two weeks postoperation, the average residual deformity angle was 3.3°, which indicates an accurate correction [28].

Several case reports and series reported similar clinical outcomes by using intraoperative aids such as 3D-printed cutting jigs, guides for drills and wires, and templates for bone grafting. The use of 3D-printed drill and cutting guides for intraoperative aids has been successfully applied to both extraarticular [30,31,32] and intraarticular [30,33,34] distal radius fracture malunion correction, malunion of metaphyseal [35] and diaphyseal [36] radius, diaphyseal ulna [36], distal humerus [30], and metacarpal bone [31], and even in nonunion correction of distal humerus [30] and epiphysiodesis correction of distal radius [37]. In malunion correction of proximal ulna, a 3D-printed navigation tool was successfully applied to obtain an accurate reposition of the ulna [38]. Furthermore, 3D-printed intraoperative aids were used as a navigation template for an accurate chondrosarcoma resection in the scapula [15] as well as for bone grafting in posttraumatic glenoid reconstruction [31] or for scaphoid reconstruction [39]. None of these studies reported 3D-printing-related adverse events, indicating that the use of 3D-printed intraoperative aids seems to be safe, especially for the use for drilling and cutting.

### 4.4. Patient-Specific Implants, Prostheses, and Orthoses

In the given review, the majority (53%, 27 of 51 studies) of the included studies reported 3D-printed implants, prostheses, and orthoses in upper extremity surgery. Again, most studies were case reports or series (24 out of 27 studies). Two RCTs [20,21] and one retrospective study [19] were identified through the conducted literature search. Kim et al. evaluated the outcome of 3D-printed hand orthoses in 22 patients with overuse syndrome of the wrist within one week, while the control group was treated with a cock-up orthosis [21]. Patients’ satisfaction was significantly higher with customized 3D-printed orthoses. However, pain relief and disability in activities of daily living did not differ between the two groups [21]. Of note, patients wore the wrist orthoses for one week only. According to this short period of time, the comparison regarding the feasibility of 3D-printed orthoses and conventional manufactured orthoses for permanent use was not possible and is still unknown due to the lack of studies [21]. Additionally, the authors highlighted that the custom 3D-printed orthoses were worn 7 h longer per day compared to cock-up orthoses, indicating better comfort. Moreover, translucent 3D-printed orthoses allow for direct monitoring of the skin [21]. While the duration of the 3D-printing process was considered as a limitation in most studies, 3D manufacturing of orthoses is in total less time-consuming compared to conventional individualized wrist orthoses with a cast molding technique. This approach takes roughly a week for production, but 3D printers may manufacture custom orthoses in just one day, and in this study within six hours [21]. The total costs, approximately USD 70 for one wrist orthosis, were similar in both groups, while customized 3D-printed orthoses were associated wither higher patient satisfaction [21]. Similar results regarding patients’ satisfaction could be found in Chen’s three-armed RCT with conservatively treated nondisplaced forearm fractures [20]. Here, satisfaction scores were significantly higher for the 3D-printed orthosis group (8.65 ± 1.040) compared to the conventional orthosis group (8.10 ± 1.252) and plaster cast (6.85 ± 1.137). Further, pain, range of motion, grip strength, and return to activity were assessed. The 3D-printed orthosis group scored significantly better (85% had good/excellent results) compared to the conventional orthosis group (70%) and plaster cast group (65%) [20]. Moreover, the complication rate of the 3D-printed group was significantly lower compared to the plaster cast and conventional orthosis groups [20]. Both RCTs [37,38], and two case reports [40], emphasize that customized 3D-printed orthoses may be an appropriate alternative in the conservative treatment of wrist pain [21] and nondisplaced forearm fractures [20,40]. In a retrospective comparative study with 30 patients, the functional outcome and complication rate of two different reconstruction methods, namely osteoarticular allograft and 3D-printed endoprosthesis, were assessed after extensive en bloc resection of giant cell tumor of the distal radius [19]. After the follow-up of 33 months, wrist function was significantly better in the 3D-printed prosthesis group compared with the conventional allograft reconstruction. Postoperative pain relief and complications were comparable in both groups. This patient-specific and anatomy-imitating endoprosthesis has shown promising results in osteoarticular reconstructions of complex biomechanical sites such as the distal radius [19].

Several case reports and series reported satisfying clinical outcomes when treating traumatic injuries, congenital diseases, and carcinoma-related resections of mostly long bones, using 3D-printed custom-made implants, prostheses, and orthoses. These 3D-printed implants have been successfully applied after extensive resection of affected bones. In giant cell tumor, en bloc resection of the affected bone is mandatory, leading to a huge bone defect to be reconstructed. A 3D-printed, patient-specific implant replacement following giant cell tumor of the distal radius and proximal phalanx was feasible and resulted in satisfying functional outcomes [41,42]. Similar results were reported for patients with osteosarcoma in the humerus [43], leiomyosarcoma of the radius [43], and chondrosarcoma of the scapula [44,45] or the humerus [46]. Patients with advanced Kienböck disease treated with an os lunatum excision followed by a titanium lunate replacement showed significantly improved wrist function and reported remarkable pain relief [47,48]. Several case reports have shown that customized 3D-printed implants, such as for the replacement of a nonunited scaphoid [49], a nonunited distal humerus [50], or the glenoid [51], are a safe and efficient alternative for the treatment of trauma-related injuries. In a case series of five patients, safety and efficacy of titanium ostheosyntheses plates for forearm osteotomies were evaluated [36] and, likewise, in a case of painful pseudarthrosis in the distal humerus [30]. Since standard ostheosynthesis plates generally do not contour directly to bony surfaces after deformity correction, the use of patient-specific plates can facilitate implant positioning [36]. Additionally, 3D-printed radial head implants seem to be a potential treatment alternative for an irreparable radial head in chronic elbow instability [52]. Chen et al. reported a successful treatment of a patient suffering from chronic clavicle osteomyelitis by clavicle replacement using a 3D-printed polyether–ether–ketone implant, which provides a promising alternative due to its antibacterial properties [53]. Further, 3D-printed custom-made prostheses may also become more important in trauma-related and congenital malformations of the hand. After partial finger [54,55] or hand amputation [56,57], customized prostheses supported daily activities, and showed an improved functional outcome, while no additional surgeries were needed.

Zuniga et al. designed a low-cost 3D-printed hand prosthesis called “Cyborg beast” for children having congenital or trauma-related hand malformations [58]. This prosthesis could be of particular interest for patients from developing countries or rural areas, as it can be adjusted remotely [59]. Nevertheless, efficacy and effectiveness, as well as the clinical outcomes for remote fitting procedures, of the 3D-printed “Cyborg beast” have not yet been investigated [58,59]. In a case series of five adolescents with congenital hand amputation, the functionality of the “Cyber beast” was assessed; all patients achieved lower scores in the evaluated tasks such as moving and holding objects [60]. The authors emphasized that the included patients were already skilled in their hand function due to their activities of daily living. The hand prosthesis may have had a negative impact on the fine motoric skills, resulting in lower functional scores [60]. Taken together, patient-specific 3D-printed implants appear to be a good option for the treatment of trauma-related and oncological issues as a means of primary bone reconstruction or as a limb-salvage procedure after conventional surgical or conservative treatment failed.

## 5. Conclusions

The clinical application of 3D printing in extremity surgery shows great potential for personalized clinical applications especially in trauma or cancer-related reconstruction. Three-dimensional printing can be used to address various challenges such as bone deformities, congenital anomalies, osteonecrosis, and overuse syndrome in various anatomical regions of the upper extremity. Three-dimensional printing has primarily been used for intraoperative templates, body implants, and preoperative planning. Benefits suggested by some authors included improved functionality and benefits in perioperative management (operation time, blood loss) without further available evidence from randomized controlled trials. The most reported outcomes were on function and pain by a variety of methods/questionnaires limiting a standardized outcome evaluation in this review, and the results were obtained from merely twelve controlled studies. With the use of a broad search strategy, we only identified twelve controlled clinical studies out of the initial 4289 studies, highlighting the lack of high-quality studies investigating 3D printing in upper extremity surgery. Studies on long-term safety and efficacy are also lacking. Based on our results, we suggest the use of standardized functional outcome parameters (e.g., “disabilities of arm, shoulder, and hand” scores, range of movement measurements before/after surgery) and pain-related parameters in long-term follow-up periods. Personalized 3D-printing tools in upper extremity surgery can be valuable assets to improve perioperative management and functional outcomes but need to be confirmed in larger properly designed clinical trials with long-term follow-up.

## 6. Limitations

This review is limited by its exclusion of publications other than in the English or German language and the search of only two databases (*PubMed* and *Web of Science*). This leaves the risk of studies being missed. Only 12 out of the 51 included studies investigated 3D-printing application in a controlled clinical setting, they had small cohorts, and they had only a short or no follow-up period. The other included 39 were case reports or series. The results presented in this review offer therefore a limited scope and should be interpreted with the relevant caution.

## Figures and Tables

**Table 1:** Operation time and intraoperative blood loss of included comparative clinical studies; 3D = three-dimensional; RCT = randomized controlled trial.

Author	Study	Indication	Number of Patients (3D: Conventional)	Clinical Application	3D Group (Mean Operative Time in Minutes; Mean Intraoperative Blood Loss in mL)	Conventional Group (Operative Time in Minutes)	*p*-Value
Yang et al. [11]	RCT	Elbow fracture	40 (20:20)	Preoperative planning	61 35.6	82 52.1	0.023 <0.001
Chen et al. [12]	RCT	Intraarticular radius fracture	48 (23:25)	Preoperative planning	66.5 41.1	75.4 54.2	<0.001 <0.001
Kong et al. [13]	RCT	Intraarticular radius fracture	32 (16:16)	Preoperative planning	51.4 52.3	63.5 74.2	<0.001 <0.001
Zheng et al. [14]	RCT	Intraarticular humerus fracture	91 (43:48)	Preoperative planning	76.6 231.1	92.0 278.6	<0.0001 <0.0001
Zhang et al. [15]	Retrospective	Cubitus varus deformity	25 (14:11)	Intraoperative aid	48.3 35.6	73.5 52.1	<0.001 <0.001
Yin et al. [16]	RCT	Scaphoid fracture/nonunions	16 (8:8)	Intraoperative aid	69.4	94.1	0.012
Schweizer et al. [17]	Prospective	Scaphoid fracture/nonunions	22 (9:13)	Intraoperative aid	118	150	0.01
Bauer et al. [18]	Retrospective	Forearm malunions	56 (25:31)	Intraoperative aid	108	140	<0.05

**Table 2:** Functional outcome of included comparative clinical studies; 3D = three-dimensional; RCT = randomized controlled trial; MEFS = Mayo Elbow Function Score; MWS = Mayo Wrist Score; VAS = Visual Analogue Scale; DASH = disability of the arm, shoulder, and hand; MMS = Modified Mayo Score; GOBS = Green and O’Brien Score; JHFT = Jebsen Hand Function Test; PRWE = Patient-Rated Wrist Evaluation.

Author	Study	Indication	Number of Patients (3D: Conventional)	Clinical Application	Test	3D Group	Conventional Group	*p*-Value
Yang et al. [11]	RCT	Elbow fracture	40 (20:20)	Preoperative planning	MEFS	88.0	82	0.001
Wang et al. [19]	Retrospective	Carcinoma (giant cell tumor)	30 (15:15)	Body implant	MWS VAS	65 1.2	71.0 1.3	0.013 0.806
Kong et al. [13]	RCT	Intraarticular radius fracture	32 (16:16)	Preoperative planning	DASH VAS	23.8 0.9	24.5 0.9	0.80 0.91
Zheng et al. [14]	RCT	Intraarticular humerus fracture	91 (43:48)	Preoperative planning	MEFS	85.2	83.1	0.448
Chen et al. [20]	RCT	Forearm fractures	60 (20:20:20)	Orthosis	GOBS	85	65.0/70	0.014/0.035
Yin et al. [16]	RCT	Scaphoid fracture/nonunions	16 (8:8)	Intraoperative aid	MMS PRWE VAS	9.4 −11.6 −4.2	5.6 −16.7 −4.17	0.52 0.52 0.98
Kim et al. [21]	RCT	Wrist pain	22 (11:11)	Orthosis	JHFT PRWE	4.3 19.2	1.0 23.4	0.101 0.109

## Data Availability

Detailed data supporting the results are available from the authors.
